# Editorial: Challenges associated with identifying preclinical animal models for the development of immune-based therapies

**DOI:** 10.3389/fimmu.2024.1404085

**Published:** 2024-03-28

**Authors:** Kainat Jamil, Shardulendra P. Sherchand, Rajan P. Adhikari, An Coosemans

**Affiliations:** ^1^ Department of Oncology, Laboratory of Tumor Immunology and Immunotherapy, Katholieke Universiteit (KU) Leuven, Leuven, Belgium; ^2^ Bacteriological Immunotherapy, AbVacc Inc., Rockville, MD, United States

**Keywords:** animal, preclinical (*in vivo*) studies, immunotherapy, immune-based therapeutics, editorial

Immunotherapy holds great potential as a cornerstone of cancer treatment due to effective clinical outcomes. Ongoing research aims to enhance its efficacy and overcome its associated pitfalls through preclinical research in animal models. However, identifying a suitable preclinical animal model, to mimic the intricate network of biological mechanisms in humans, is challenging (Chen et al.). The same holds true for autoimmune diseases, like rheumatoid arthritis (RA), inflammatory bowel disease (IBD), collagen-induced arthritis (CIA), Crohn’s disease, systemic lupus erythematosus (SLE), reactive arthritis, type I diabetes, juvenile arthritis and sclerosis. Findings from pre-clinical animal models have the potential to be extrapolated to humans, but the question is if a single animal model can help us find answers to different autoimmune diseases induced by the intricate interplay between host, microbes, and autoimmune response.

This was perfectly underscored by the publications of Berckmans et al. and Sitnikova et al. Both researchers demonstrated that the choice of the preclinical model is crucial to accurately reflect the clinical situation and draw meaningful conclusions from preclinical therapy testing. Given the fact that it is of utmost importance that these models have an intact immune system, also age and realistic location of the tumor (orthotopic models) are needed. Sitnikova et al. investigated how aging can impact the immune profile and tumor progression through their experimental research in 60-72 weeks-old mice models. They observed reduced naïve T cell populations and fewer intratumoral CD8 T cell versus T regulatory (CD8/Treg) cell fractions in aged mice compared to young mice. On average, since cancer is mostly diagnosed in older people, the authors recommend to extend preclinical research in aged mice to predict accurately the treatment response. Furthermore, Giardino Torchia and Moody developed a framework called DIAL, to characterize the testing of cell therapies in mice. The DIAL model evaluates the current preclinical evidence at four levels (Distribution, Infiltration, Accumulation, and Longevity) and highlights which preclinical models could answer the research question (= on gene-modified lymphocyte therapy development) best.

In addition, it is important to consider the distinct time points during tumor progression to evaluate the immune profile in preclinical models. Shields et al. provided a comprehensive overview of the MC38 colorectal cancer preclinical model utilized in investigating T cells during tumor progression to understand the immune-resistance mechanisms. The authors underscored the importance of time-point selection in elucidating the disparities in early-stage and late-stage tumor microenvironments (TME). They demonstrated that the early-stage tumors displayed primitive TME, lacking clinically relevant immune-resistance mechanisms. In contrast, the late-stage MC38 tumor model depicted a mature and developed TME like human TME, characterized by T cell exhaustion, T cell exclusions, and desmoplasia.

Zhou et al. reviewed the advancements achieved with human tumor models in mice and rats. They compared different mice models (induced, spontaneous, transgenic, transplantable, patient-derived xenografts (PDX), and humanized mice models) and explored the development processes along with the advantages and disadvantages. The success rate of implanting human cells in mice has been constantly low, from the earliest nude mice and progressing to the later severe combined immunodeficiency (SCID) and nonobese diabetic/severe combined immunodeficiency (NOD/SCID) mice. All humanized mice models lack one or more immune cells, limiting the human immune system reconstruction in animal models. The recent humanized-hematopoietic stem cells (Hu-HSCs) model with less graft-versus-host disease (GvHD) is expected to be used for long-term research, however, it is still lacking human cytokines and stem cells.

Jia et al. went for another approach. They demonstrated that Syrian hamsters exhibit characteristics like humans in terms of their anatomy, physiology, and pathology and can act as the preferred emerging tumor model to develop immunotherapies. They further reported that human granulocyte-macrophage colony-stimulating factor (GM-CSF), human interleukin (IL)-21 and IL-12 are immunologically active in this tumor model. However, the absence of specialized immune reagents poses a challenge in conducting thorough investigations. Initiatives are ongoing to address these limitations by establishing repositories of Syrian hamster tumor cell lines, designing transgenic hamster models, and producing research reagents to precisely evaluate immune functionalities.

Similarly, Xu et al. focused on establishing an orthotopic tumor model in C57BL/6 mice to better understand the immune biology of and validate new therapeutic targets for anaplastic thyroid cancer. The research group compared the original B6129SF1/J hybrid mice model with the adapted model by molecular characterization of the tumor and immune cell populations. They observed upregulation of both pro-inflammatory cells (cytotoxic T cells, helper T cells, pro-inflammatory M1-macrophages) and immune suppressive cells (myeloid-derived suppressor cells), along with enhanced proliferative characteristics of cancer cells. However, the *in vitro* and *in vivo* results were contradictory regarding tumor growth. Therefore, further research in this new model is warranted before it can be further used.

Differences between anatomy, histology, and sites of prostatitis in men and rodents are reviewed by He et al. Inflammation pertaining to prostatitis could be acute, chronic, due to a pathogen infection or non-infectious immune-related. For all these subtypes, various preclinical models exist, e.g., non-bacterial prostatitis models arise from hormonal disorders, stress, urinal reflux, nervous dysfunction chemicals, immune mediations diet, and lactations. Readouts for animal models of prostatitis could be changes in behavior, body weight, pathology of the prostate, biochemical results, and measurements of urodynamics. This study highlights the complexity to study prostatitis and it underscores the importance of using multiple animal models to study the disease.

Karmele et al. reviewed single-cell RNA-sequencing (scRNA-seq) similarities and differences between human and animal IBD. There is a complex interaction between immune, microbiological, stromal and epithelial cells. In this study, they also summarized common pre-clinical models of IBD with various mechanisms and their advantages over other models. Chronic inflammation of intestinal cells can be induced chemically, by genetically knocking out IL10, antibody-mediated, via bacterial infections or spontaneously. Destruction of intestinal cells can be generated chemically and genetically by trinitrobenzene sulfonic acids, oxazolone and IL10 knockouts. Spontaneous colitis models can be developed by the production of TNFα, IL23, and Th1 driven immune response in AKR mice, who spontaneously develop lymphatic leukemia. Interestingly, infection of murine intestinal epithelial cells with *Citrobacter rodentium* and *Helicobacter hepaticus* can activate cytokine production and induce inflammation which serves as a good pre-clinical model for studying IBD (1).

Likewise, Kim et al. identified RA-associated beneficial microbial species; out of 36 isolates they found *Peptoniphilus gorbachii* to have major significance. Their results showed that *P. gorbachii* alleviated CIA in mice by improving intestinal homeostasis and immune regulations. Their novel findings in CIA mice were confirmed by low serum antibody levels against *P. gorbachii* in patients with worse disease outcomes.

Another successful use of animal models is the mechanically ventilated rabbit model of hyperdynamic septic shock. Nguyen et al. showed that passive immunization with neutralizing alpha-hemolysin, biocomponent leukocidins, and clumping factor A has protective efficacy against *Staphylococcus aureus* mediated acute septic shock in the mechanically ventilated rabbit model. In this model, authors measured cardiac output (CO), stroke volume (SV), and systemic vascular resistance (SVR). Mean arterial pressure (MAP) decline, increased SVR, reduced SV, increased CO, and reduced left ventricular ejection volume could be measured in this rabbit model. These parameters mimicked the clinical hallmarks of septic shock triggered by *S. aureus* in patients.

Besides the relevance of a clinical representative immune biology mimicked in the preclinical models, the evaluation of treatments is of course the true reason why we perform preclinical experiments. Berckmans et al. also demonstrated that the timing of the treatments and their order will influence the survival of the mice and that this is (most of the time) not considered when designing preclinical and clinical trials. Kametani et al. evaluated liposome-encapsulated anti-programmed death ligand 1 antibody-conjugated progesterone in humanized peripheral blood mononuclear cells (Hu-PBMCs) breast cancer mice models. The authors noticed that GvHD and short survival periods can affect the evaluation of long-term immune response. Therefore, additional research is required. Gao et al. evaluated the effects of transdermal formestane anti-breast cream using a rat model. They induced mammary carcinoma in female Sprague-Dawley rats using dimethylbenz(a)anthracene administrations. Results demonstrated downregulation of extracellular matrix-related genes, increased immune cell infiltration in tumors, and negative effects on cell growth. The study suggests that formestane can potentially modulate the tumor microenvironment and immune response in breast cancer leading to improved outcomes. Kyuuma et al. developed nanobodies (ozoralizumab) against TNFα that lack the Fc portion of IgG, abrogating immune complex-induced inflammation in an RA model of mice compared to adalimumab (a TNFα inhibitor).

Animal models are very important surrogates for humans to measure pharmacodynamics, toxicity, immunogenicity, biomarkers, and allergic reactions to therapeutics. However, efficacy data generated by animal models are hard to reproduce in humans. The enormous repertoire of preclinical models already available and the continuous creation of newer models should provide us in the future with dedicated models to answer specific research questions. Nevertheless, some parts are still understudied/underrepresented, like for example the response of the nervous system. Humans are a highly developed intellectual entity, and it is understandable that our neuronal response to host and pathogen interaction is different than other animal models which are severely understudied. It is of utmost importance to consider different aspects of a preclinical animal model for evaluating the efficacy of immune-based therapies to translate into better clinical outcomes.

## Author contributions

KJ: Writing – original draft, Writing – review & editing. SS: Writing – original draft, Writing – review & editing. RA: Writing – original draft, Writing – review & editing. AC: Writing – original draft, Writing – review & editing.
